# Role of multidetector computed tomography in the diagnosis and management of patients attending the rapid access chest pain clinic, The Scottish computed tomography of the heart (SCOT-HEART) trial: study protocol for randomized controlled trial

**DOI:** 10.1186/1745-6215-13-184

**Published:** 2012-10-04

**Authors:** David E Newby, Michelle C Williams, Andrew D Flapan, John F Forbes, Allister D Hargreaves, Stephen J Leslie, Steff C Lewis, Graham McKillop, Scott McLean, John H Reid, James C Sprat, Neal G Uren, Edwin J van Beek, Nicholas A Boon, Liz Clark, Peter Craig, Marcus D Flather, Chiara McCormack, Giles Roditi, Adam D Timmis, Ashma Krishan, Gillian Donaldson, Marlene Fotheringham, Fiona J Hall, Paul Neary, Louisa Cram, Sarah Perkins, Fiona Taylor, Hany Eteiba, Alan P Rae, Kate Robb, Dawn Barrie, Kim Bissett, Adelle Dawson, Scot Dundas, Yvonne Fogarty, Prasad Guntur Ramkumar, Graeme J Houston, Deborah Letham, Linda O’Neill, Stuart D Pringle, Valerie Ritchie, Thiru Sudarshan, Jonathan Weir-McCall, Alistair Cormack, Iain N Findlay, Stuart Hood, Clare Murphy, Eileen Peat, Barbara Allen, Andrew Baird, Danielle Bertram, David Brian, Amy Cowan, Nicholas L Cruden, Marc R Dweck, Laura Flint, Samantha Fyfe, Collette Keanie, Tom J MacGillivray, David S Maclachlan, Margaret MacLeod, Saeed Mirsadraee, Avril Morrison, Nicholas L Mills, Fiona C Minns, Alyson Phillips, Laura J Queripel, Nicholas W Weir, Fiona Bett, Frances Divers, Katie Fairley, Ashok J Jacob, Edith Keegan, Tricia White, John Gemmill, Margo Henry, James McGowan, Lorraine Dinnel, C Mark Francis, Dennis Sandeman, Ajay Yerramasu, Colin Berry, Heather Boylan, Ammani Brown, Karen Duffy, Alison Frood, Janet Johnstone, Kirsten Lanaghan, Ross MacDuff, Martin MacLeod, Deborah McGlynn, Nigel McMillan, Laura Murdoch, Colin Noble, Victoria Paterson, Tracey Steedman, Nikolaos Tzemos

**Affiliations:** 1 University of Edinburgh/BHF Centre for Cardiovascular Science, Chancellor’s Building, 49 Little France Crescent, Edinburgh, EH16 SU4, UK; 2 Royal Infirmary of Edinburgh, 51 Little France Crescent, Edinburgh, EH16 4SA, UK; 3 School of Economics, University of Edinburgh, 31 Buccleuch Place, Edinburgh, EH8 9JT, UK; 4 Forth Valley Royal Hospital, Larbert, FK5 4WR, UK; 5 Raigmore Hospital, Inverness, IV2 3UJ, UK; 6 Edinburgh Clinical Trials Unit, University of Edinburgh, Western General Hospital, Crewe Road South, Edinburgh, EH4 2XU, UK; 7 Barts Health NHS Trust, The Royal London Hospital, London, E1 1BB, UK; 8 Borders General Hospital, Melrose, TD6 9BS, UK; 9 Clinical Research Imaging Centre, University of Edinburgh, Queen’s Medical Research Institute, 47 Little France Crescent, Edinburgh, EH16 4TJ, UK; 10 Peninsula Heart & Stroke Network, Plymouth, PL6 5QZ, UK; 11 Chief Scientist Office, Scottish Government Health Directorates, St Andrew’s House, Edinburgh, EH1 3DG, UK; 12 Royal Brompton Hospital, Sydney Street, London, SW3 6NP, UK; 13 Glasgow Royal Infirmary, 16 Alexandra Parade, Glasgow, G31 2ER, UK; 14 Western Infirmary, Glasgow/Institute of Cardiovascular & Medical Sciences, University of Glasgow, Glasgow, G12 8QQ, UK; 15 London Chest Hospital, Bonner Road, London, E2 9JX, UK; 16 Ninewells Hospital, Dundee, DD1 9SY, UK; 17 Royal Alexandra Hospital, Paisley, PA2 9PN, UK; 18 Western General Hospital, Edinburgh, EH4 2XU, UK; 19 St John’s Hospital, Livingston, EH54 6PP, UK; 20 University Hospital, Ayr, KA8 0RX, UK; 21 Victoria Hospital, Kirkcaldy, KY2 5RA, UK

**Keywords:** Computed tomography, Coronary heart disease, Rapid access chest pain clinic

## Abstract

**Background:**

Rapid access chest pain clinics have facilitated the early diagnosis and treatment of patients with coronary heart disease and angina. Despite this important service provision, coronary heart disease continues to be under-diagnosed and many patients are left untreated and at risk. Recent advances in imaging technology have now led to the widespread use of noninvasive computed tomography, which can be used to measure coronary artery calcium scores and perform coronary angiography in one examination. However, this technology has not been robustly evaluated in its application to the clinic.

**Methods/design:**

The SCOT-HEART study is an open parallel group prospective multicentre randomized controlled trial of 4,138 patients attending the rapid access chest pain clinic for evaluation of suspected cardiac chest pain. Following clinical consultation, participants will be approached and randomized 1:1 to receive standard care or standard care plus ≥64-multidetector computed tomography coronary angiography and coronary calcium score. Randomization will be conducted using a web-based system to ensure allocation concealment and will incorporate minimization. The primary endpoint of the study will be the proportion of patients diagnosed with angina pectoris secondary to coronary heart disease at 6 weeks. Secondary endpoints will include the assessment of subsequent symptoms, diagnosis, investigation and treatment. In addition, long-term health outcomes, safety endpoints, such as radiation dose, and health economic endpoints will be assessed. Assuming a clinic rate of 27.0% for the diagnosis of angina pectoris due to coronary heart disease, we will need to recruit 2,069 patients per group to detect an absolute increase of 4.0% in the rate of diagnosis at 80% power and a two-sided *P* value of 0.05. The SCOT-HEART study is currently recruiting participants and expects to report in 2014.

**Discussion:**

This is the first study to look at the implementation of computed tomography in the patient care pathway that is outcome focused. This study will have major implications for the management of patients with cardiovascular disease.

**Trial registration:**

ClinicalTrials.gov Identifier: NCT01149590

## Background

The clinical presentation of chest pain is a major problem for primary health care professionals and is the commonest medical reason for a patient attending the Emergency Department. Ascertaining the aetiology of the chest pain is essential not only for the future management and investigation of the patient, but also for health care resources to be utilized appropriately and efficiently. The distinction between cardiac and noncardiac chest pain can be subtle, leading, in some series [1,2], to between 2 and 12% of patients being inappropriately discharged from hospital and more than 25% being readmitted to hospital with benign noncardiac chest pain. From the primary care perspective, Emergency Department attendances or short-term hospitalizations with an unhelpful diagnosis, such as ‘chest pain - myocardial infarction excluded’, do not provide a clear diagnosis or management plan.

### Rapid access chest pain clinics

The accurate identification of patients with ischaemic heart disease is important because up to 30% of patients presenting with recent-onset angina have a cardiac event within 1 to 2 years [3] and many of these patients may benefit from coronary revascularization [4]. This has led many centres to develop the provision of a rapid access chest pain clinic. This out-patient clinic provides a ‘one-stop’ assessment for patients with suspected angina, including medical history, examination, electrocardiogram, blood tests, and exercise testing where appropriate. It does not include patients with acute chest pain who require immediate hospital assessment for suspected acute coronary syndrome. We have demonstrated that such services reduce the hospitalization of patients with benign noncardiac chest pain whilst facilitating the identification of those patients with acute coronary syndromes requiring in-patient care [5]. A specialist cardiology opinion combined with the resources of a chest pain clinic service would appear to have a higher diagnostic yield for ischaemic heart disease than open-access exercise electrocardiography, and would provide the primary care physician with a firm clinical diagnosis in the majority of cases, and identify those patients requiring further invasive investigation [5].

### The need for better diagnostic accuracy and risk stratification

Rapid access chest pain clinics have now become established across the United Kingdom and they have proven successful in identifying high-risk patients with coronary heart disease [6]. However, there is room for improvement, with some patients continuing to be misdiagnosed with noncardiac chest pain [6]. Moreover, those diagnosed with noncardiac chest pain account for up to a third of patients who subsequently die from cardiovascular disease or suffer an acute coronary syndrome over 5 years of follow-up [6]. There is, therefore, a need for better diagnostic accuracy and risk stratification in patients attending rapid access chest pain clinics, especially in younger patients (<65 years) [7].

### Coronary artery calcification

Coronary artery calcification is an independent risk factor for coronary heart disease, with even low coronary calcium scores doubling the risk of coronary events [8]. The relative risk associated with coronary calcification is greater than that associated with established factors, such as smoking, hypertension and diabetes mellitus. The progression of coronary artery calcification is associated with a higher incidence of coronary events, even in those people who are asymptomatic at the time of initial scanning [9]. Thus, the presence of coronary artery calcification is not only indicative of atheromatous plaque disease, but its progression may correspond with cardiovascular event rates.

The degree of calcification correlates with atheroscle rotic burden but it does not identify soft plaque and may not predict the patient’s response to medical interventions [10,11]. Moreover, the presence of coronary artery calcification does not, in itself, predict the presence of obstructive atheroma. Calcification can, therefore, be used as a surrogate marker of the extent of coronary atherosclerotic disease, rather than as a measure of luminal stenosis [12].

### Computed tomography coronary angiography

Major advances in scanning technology have led to the establishment of noninvasive coronary angiography by multidetector computed tomography (MDCT). This has a very good agreement with invasive coronary angiography [13,14] and intravascular ultrasound [13-16], with kappa coefficient values of 0.75 for both. The resolution of modern scanners allows quantification of luminal stenoses as well as identification of noncalcified ‘soft’ atherosclerotic plaque [15]. Pooled analysis of over 800 patients indicates a sensitivity of 89% (95% confidence intervals, 87 to 90%) and specificity of 96% (95% confidence intervals, 96 to 97%) for 64-MDCT in comparison with invasive coronary angiography [16]. The major strength is in the negative predictive value of 98% (95% confidence intervals, 98 to 99%). The current evolution of scanning technology has led to greater spatial and temporal resolution with lower radiation doses (~2 to 3 mSv). This should translate into a highly effective and safe imaging strategy, particularly for the evaluation of stable patients with possible coronary artery disease [17].

It is important to highlight that computed tomography (CT) coronary angiography is primarily used to confirm or refute the presence of coronary artery disease. The diagnosis of angina pectoris due to coronary heart disease primarily relies on two factors: (i) a history consistent with angina pectoris, and (ii) the presence of obstructive coronary artery disease. It should be realized that patients with coronary heart disease may have nonanginal chest pain and that patients with typical anginal chest pain may not have coronary heart disease (see Table 1).

**Table 1:** Categorization of patients based on presence the presence or absence of angina pectoris and coronary heart disease

		**Coronary heart disease**	
		**Yes**	**No**
**Angina pectoris**	**Yes**	High-risk and cause of symptoms	Low-risk and cause of symptoms unclear
	**No**	High-risk but either symptoms unrelated or atypical presentation	Low-risk and other cause of symptoms likely

A health technology assessment including a comprehensive systematic review of 64-multidetector CT coronary angiography highlighted several areas that require further research, including (i) the usefulness of MDCT coronary angiography in patients with suspected coronary artery disease; (ii) the advantages of 256- versus 64-MDCT coronary angiography; and (iii) the role of MDCT to assess coronary artery plaque morphology [18]. In addition, the National Institute of Clinical Excellence (NICE) specifically called for research into the clinical efficacy and cost-effectiveness of MDCT coronary angiography compared with functional testing in the diagnosis of angina [19].

## Methods/design

### Study design

This is an open parallel group prospective randomized controlled trial, assessing the impact of CT on the diagnosis and management of patients attending a rapid access chest pain clinic.

### Study objectives

The purpose of a rapid access chest pain clinic is to identify patients with symptoms of angina attributable to coronary heart disease, in order to identify those who would benefit from secondary prevention and anti-anginal therapies. The standard approach is to document a clinical history of angina pectoris and demonstrate objective evidence of exercise-induced myocardial ischaemia through exercise stress testing. We wish to evaluate the added value of coronary artery calcium scoring and CT coronary angiography in the assessment of patients attending a rapid access chest pain clinic.

In the setting of a rapid access chest pain clinic, the most important question that the patient has is whether his or her chest pain is due to coronary heart disease. The primary objective of the study, therefore, is to investigate whether the inclusion of coronary artery calcium scoring and CT coronary angiography alters the proportion of patients diagnosed with angina due to coronary heart disease at 6 weeks.

The secondary objectives of the study are to ascertain whether a coronary artery calcium score and CT coronary angiogram influences the management of patients with coronary heart disease or noncardiac causes of chest pain. Patients’ concerns often relate to the chest pain itself (symptoms), what causes their symptoms (diagnosis), what further tests are required (investigations), what medication or procedures are recommended (treatments) and what the impact will be on their future health (long-term outcomes). We will also assess safety and health economic outcomes. We will undertake long-term follow of these patients to determine whether CT assessments independently predict future risk, and whether this leads to improved clinical outcomes through better guided use of therapies.

### Primary endpoint

The primary endpoint of the study will be the proportion of patients diagnosed with angina pectoris secondary to coronary heart disease at 6 weeks. The clinician in charge of the patients’ care will assign the diagnosis following either (a) analysis of the coronary artery calcium score and CT coronary angiogram (intervention group), or (b) standard care (conservative group).

### Secondary endpoints

The following secondary endpoints will be evaluated: (i) the frequency and severity of chest pain symptoms at six weeks and six months; (ii) the CT observed presence and extent of coronary artery disease; (iii) the diagnosis and severity of coronary heart disease; (iv) the accuracy of CT coronary angiography compared with the gold-standard of invasive coronary angiography in those who receive both investigations; (v) the effect on investigations, unscheduled care and healthcare resource utilization; and (vi) the effect on patient management, including secondary prevention, anti-anginal therapy (pharmacological therapy and coronary revascularization), and treatment of noncardiac chest pain (such as hiatus hernia).

The study has been primarily set up to assess a patient-focused symptom outcome. However, alterations in management may result in long-term benefits to patients. Therefore long-term outcomes will be assessed, including: (i) cardiovascular death or nonfatal myocardial infarction; (ii) cardiovascular death; (iii) nonfatal myocardial infarction (universal definition); (iv) cardiovascular death, nonfatal myocardial infarction or nonfatal stroke; (v) nonfatal stroke; (vi) all causes of death; (vii) coronary revascularization, percutaneous coronary intervention or coronary artery bypass graft surgery; (viii) hospitalization for chest pain, including acute coronary syndromes and noncoronary chest pain; and (ix) hospitalization for cardiovascular disease, including coronary artery disease, cerebrovascular disease and peripheral arterial disease.

### Patient population

Participants will be identified from patients attending the rapid access chest pain clinic. We will recruit 4,138 patients, randomized 1:1 to standard care (*n* = 2,069) or standard care with coronary calcium score and CT coronary angiography (*n* = 2,069).

Inclusion criteria will be: (i) attendance at the rapid access chest pain clinic and (ii) age over 18 years but less than or equal to 75 years.

Exclusion criteria will be: (i) inability or unwillingness to undergo CT scanning; (ii) exceeding the weight tolerance of scanner; (iii) known severe renal failure (serum creatinine >200 μmol/l or estimated glomerular filtration rate <30 ml/min); (iv) previous recruitment to the trial; (v) major allergy to iodinated contrast agent; (vi) inability to give informed consent; (vii) known pregnancy; or (viii) acute coronary syndrome within 3 months.

### Participant selection and enrolment

The trial is a pragmatic evaluation of the added value of CT coronary angiography in a rapid access chest pain clinic. Only patients attending the rapid access chest pain clinic will be approached to participate. The inclusion and exclusion criteria are broad and inclusive, and should enable rapid identification of eligible patients attending the clinic.

**Figure 1:**
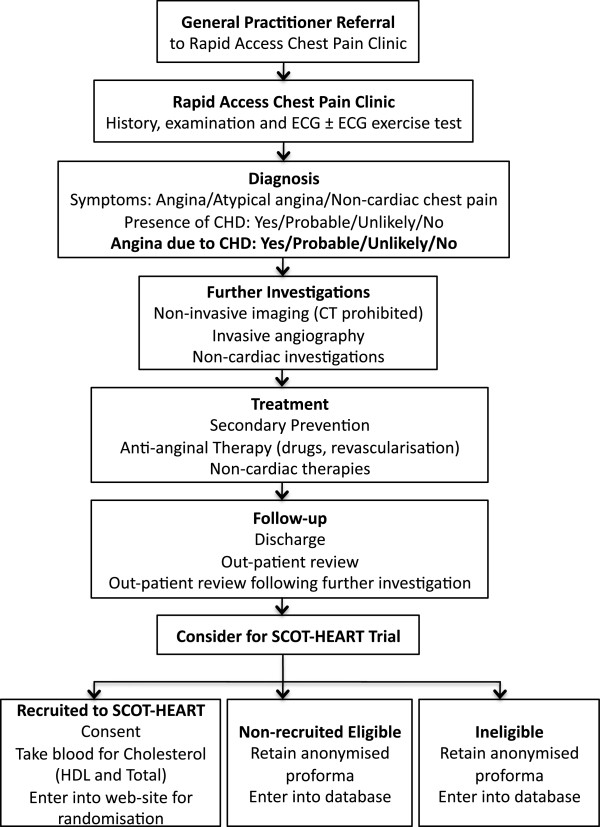
**Rapid access chest pain clinic attendance, eligibility and recruitment.** (CHD, coronary heart disease; CT, computed tomography; ECG, electrocardiogram; HDL, high dependency lipoprotein).

Patients will be given a patient information sheet on arrival at the rapid access chest pain clinic. After consultation, all eligible patients will be approached to enter the trial by the attending clinician. Written informed consent will be obtained from patients willing to participate in the study (Figure 1).

### Randomization

Following recruitment, patients who agree to undergo research evaluation will be randomized (1:1), either to no additional scanning or to undergo further evaluation with coronary calcium scoring and CT coronary angiography within 14 days of clinic attendance and before invasive coronary angiography or other cardiac investigations. Randomization will be conducted using a web-based system to ensure allocation concealment and will incorporate minimization to ensure matching for age, sex, body mass index (height and weight), diabetes mellitus, prior history of coronary heart disease, atrial fibrillation and baseline diagnosis of angina due to coronary heart disease.

### Equipoise

Attending clinicians in the rapid access chest pain clinic will not be permitted to investigate patients with CT coronary angiography unless the patient is randomized to receive the scan as part of the trial. During the course of the trial, it is anticipated that CT coronary angiography may start to be introduced into routine clinical practice within the rapid access chest pain clinic setting. It will be important that loss of equipoise does not prejudice the trial. This practice will, therefore, be discouraged during the course of the trial. Where this is not feasible, either the trial centre will be closed or all patients will be approached to participate in the trial but, where a patient is randomized to standard of care, they will be excluded from undergoing a CT coronary angiogram and an alternative noninvasive test will be selected. Equipoise will be monitored through data collected on unrecruited patients.

### Study assessments and data collection

#### Clinic assessment

All patients will undergo routine evaluation at the rapid access chest pain clinic including, where appropriate, symptom-limited exercise electrocardiography using the standard Bruce protocol. Patients will be categorized as low, intermediate or high risk by the attendant clinician, informed by the NICE guideline [20]. In general, high-risk patients will be treated for coronary heart disease and undergo invasive coronary angiography (10%), and low-risk patients (10%) will be reassured and discharged. Intermediate-risk patients (80%) will be treated and further investigated at the discretion of the clinician. In all cases, the diagnosis and agreed management strategy will be documented at the end of the clinic attendance. Cardiovascular risk will be calculated using previously established risk scores, such as the ASSIGN and Framingham scores.

The study data sheet will be used in the rapid access chest pain clinic to document patient history, examination and management plan for all patients (recruited and unrecruited). This will be completed by the attending clinician or nurse. This will also include assessment of eligibility. The study pro forma for all ineligible participants and eligible unrecruited participants will be retained in an anonymized form to provide detailed data on these patients in comparison with the study participant population. All study pro formas (recruited and unrecruited eligible and ineligible patients) and consent forms (recruited participants) will be collated by the trial manager and entered into the database.

#### Blood tests

If the participant has not had total cholesterol and HDL (high-density lipoprotein) cholesterol levels measured within the past three months, a blood test will be taken at the rapid access chest pain clinic. If the total cholesterol is above 7.0 mmol/l or the HDL is below 0.5 mmol/l, the primary care physician will be informed by letter, as this may warrant treatment irrespective of the patient’s 10-year cardiovascular risk.

In a sub-group of patients, a venous blood sample will be obtained and stored for future assessment of biomarkers.

#### Computed tomography

Computed tomography scans will be performed using a 64, 128 or 320-multidetector scanner. Computed tomography protocol optimizations will be performed at all sites throughout the study, to optimize scanning parameters, such as radiation dose and contrast administration.

##### Medication

Before calcium scoring, patients with a heart rate of greater than 60 beats/min and systolic blood pressure >110 mmHg will receive rate-limiting medication. If a participant’s heart rate is above 100 beats per minute despite rate-limiting medication, CT coronary angiography will not be performed. A small dose of oral diazepam may be prescribed for anxious patients, to improve heart rate control. Sublingual glyceryl trinitrate will be administered immediately prior to CT imaging.

##### Coronary artery calcium score

Coronary calcium scoring will be performed prior to coronary angiography. Investigators blind to patient characteristics will conduct off-line analyses using automated computerized software programs that employ the Agatston scoring method [21] using a threshold of 130 Hounsfield units [11]. The calcium score percentile based on age and sex will be calculated using coronary artery calcium score distributions from the Multi-Ethnic Study of Atherosclerosis (MESA) [22]. This will be performed using a web-based calculator, available at http://www.mesa-nhlbi.org/Calcium/input.aspx. For patients younger than 45, 45 years will be used for the calculation of the calcium score percentile.

##### Computed tomography coronary angiography

Coronary angiography will be conducted during contrast enhancement using pre-specified protocols (as recommended by the scanner manufacturers) during a single breath hold with prospective electrocardiographic gating as appropriate.

### Image assessment

All CT and invasive coronary angiograms will be assessed by at least two trained observers. Angiograms (CT and invasive) will be reviewed independently and without prior knowledge of the alternate angiogram. Where there is disagreement between paired observers (but not modalities), angiograms will be reviewed and classified by consensus. Angiograms will be reported using the 15-segment model [23]. Significant stenosis due to coronary artery disease will be defined as a stenosis greater than 70% in one or more major epicardial vessels or greater than 50% in the left main stem [20]. Luminal cross-sectional area stenoses will be classified as normal (<10%), haemodynamically insignificant (10-49%), intermediate (50-70%), significant (greater than or equal to 70%) or total or subtotal occlusion (100%).

### Definition of coronary artery disease

Coronary artery disease will be classified as: (a) obstructive coronary artery disease, atherosclerotic plaque encompassing a luminal cross-sectional area of ≥70% in at least one major epicardial vessel; (b) nonobstructive coronary artery disease, either atherosclerotic plaque encompassing a luminal cross-sectional area of <70% but >10% in at least one major epicardial vessel, or a calcium score >400 AU (Agatston units) or >90th percentile for age and sex; or (c) minimal or no coronary artery disease. Significant plaque burden is defined as atherosclerotic plaque causing >10% luminal cross-sectional area stenosis.

### Management recommendations

**Figure 2:**
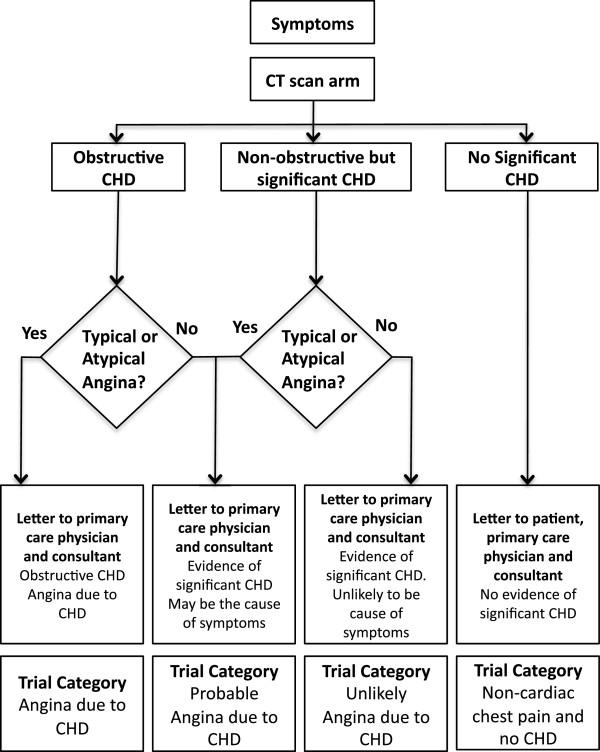
**Computed tomography coronary angiography results and diagnosis documentation.** (CHD, coronary heart disease; CT, computed tomography).

**Figure 3:**
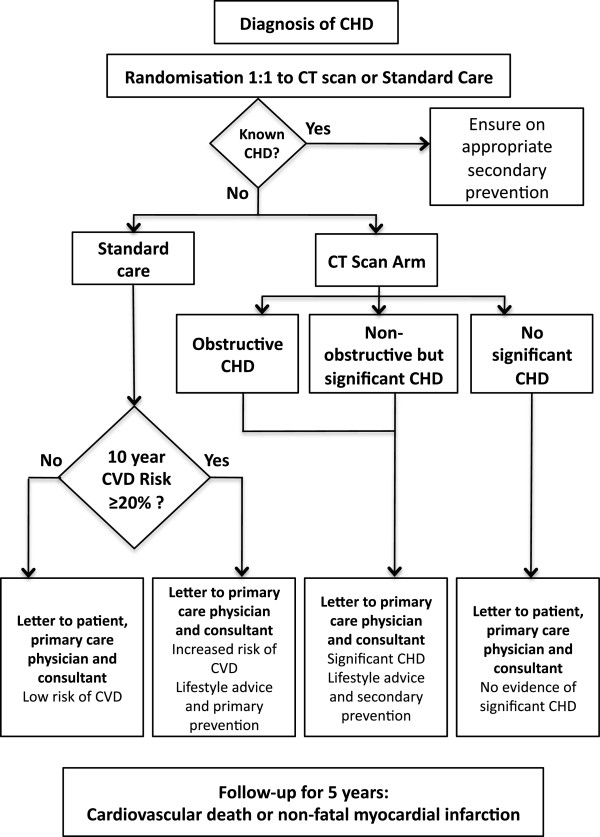
Plan of investigation (CHD, coronary heart disease; CT, computed tomography; CVD, cardiovascular disease).

Computed tomography scans will be reported locally by two trained observers (radiologist and cardiologist). The CT report will also include recommendations for management, including primary or secondary prevention. Treatment of angina due to coronary heart disease will be at the discretion of the responsible consultant. (Figures 2 and 3). For participants in the no-CT-scan group, the responsible consultant will be sent a letter detailing the patient’s ASSIGN score.

### Symptom outcomes

At baseline, 6 weeks and 6 months, chest pain will be assessed by the UK version of the Seattle Angina Questionnaire (SAQ-UK) [24] and quality of life will be assessed by the 12-Item Short Form Health Survey (SF-12®). The standard (4-week) recall second version of the SF-12® will be used (SF-12v2™). The SF-12 Physical Component Summary (PCS) and Mental Component Summary (MCS) scores will be calculated, along with utility scores based on the SF-6D algorithm.

Baseline questionnaires will be handed to the patient at the end of the rapid access chest pain clinic consultation. At 6 weeks and 6 months, questionnaires will be posted to participants, with telephone follow-up for nonresponders after two mailings two weeks apart.

### Diagnostic outcomes

The proportion of patients diagnosed with coronary heart disease will be documented at baseline, after CT (where appropriate), after 6 weeks and after 6 months. This will be defined as (i) prior history of coronary heart disease (previous documented acute myocardial infarction (universal definition), obstructive coronary heart disease (≥70% luminal stenosis in at least one major epicardial vessel on invasive coronary angiography) or previous coronary revascularization (percutaneous coronary intervention or coronary artery bypass surgery)), (ii) clinical diagnosis of angina pectoris due to coronary heart disease, or (iii) obstructive or nonobstructive CT diagnosis of coronary heart disease. The extent of coronary heart disease will be determined by the number of vessels affected (none, one, two or three vessels diseased (≥70% luminal stenosis of a major epicardial vessel)) and plaque load determined by CT coronary angiography.

The accuracy of the CT coronary angiography will be determined by comparison with invasive coronary angiography (gold-standard) for the assessment of the number of vessels affected. The extent of plaque burden cannot be compared between the two modalities.

### Investigation outcomes

Relevant investigations will be documented for each participant at the baseline clinic attendance, after 6 weeks and after 6 months. This will include: an exercise electrocardiographic stress test; nuclear medicine imaging - myocardial perfusion imaging; stress echocardiography; invasive coronary angiography; and noncardiac investigations, for example, endoscopy.

This will be documented by the clinician in charge of patient care in response to the CT coronary angiogram report. Further information will be obtained from electronic hospital records and patient surveys. Computed tomography coronary angiography will be performed prior to any invasive coronary angiogram, to facilitate a decision to undertake or cancel this invasive investigation.

### Treatment outcomes

All treatments will be documented at baseline, after CT scan (where appropriate), and at 6 weeks and 6 months. Documentation of current prescribed medications will be obtained from patients, electronic hospital records or general practitioners. This will include:

1. * Secondary prevention.* Prescription of drug therapy for the prevention of cardiovascular events will be documented; drugs listed will include aspirin, clopidogrel, other anti-platelet agents, statins, angiotensin-converting enzyme inhibitor therapy, and beta-blockers.

2. * Pharmacological anti-anginal therapy.* Prescription of drug therapy (drug, class, dose) for the alleviation of angina pectoris will be documented; this will include beta-blockades, calcium antagonists, nitrates, nicorandil, and ivabradine.

3. * Coronary revascularization.* The use of percutaneous coronary intervention and coronary artery bypass surgery will be documented at 6 weeks and 6 months.

### Long-term outcomes

Annual hospitalizations for chest pain episodes, acute coronary syndromes, coronary revascularization procedures, cerebrovascular disease and peripheral vascular disease will be recorded from the Information and Statistics Division of NHS Scotland, and deaths from the Central Registry Office, Scotland for up to 10 years following trial enrolment. Where possible, events will be corroborated by electronic hospital records and case note review.

### Diagnosis and management documentation

The CT scan report will include a section requesting the documentation of any changes in the diagnosis, investigation and treatment of the participant. The chest pain clinic nurse or responsible consultant will complete this pro forma and return it to the trial manager.

At 6 months, trial and data manager will document changes in patient diagnosis, investigation and treatment for all patients at 6 weeks and 6 months using the TrakCare™ software application (InterSystems Corporation, Cambridge, MA, USA), which is an electronic patient record system used by the National Health Service (NHS) Lothian Health Board. This system is destined to be adopted by other centres throughout Scotland. Where appropriate, this will be supplemented by source document review.

### Safety outcomes

#### Radiation dose

The main safety concerns relate to exposure to ionizing radiation [25]. The dose-length product (DLP) will be recorded and the effective radiation dose will be calculated using the conversion factor method. Age- and sex-specific lifetime attributable risks of cancer will be estimated using the Biological Effects of Ionizing Radiation VII Phase 2 report [26]. All incident cancers identified during the study will be recorded throughout the follow-up phase of the trial.

#### Incidental findings

Incidental findings occur in 22 to 74% of CT scans of the chest but only 1.7% of these are clinically significant [27]. Nevertheless, any incidental findings may require further investigations, involving exposure to ionizing radiation. An incidental finding will be defined as an abnormality identified on CT without antecedent clinical suspicion or previously known disease [27]. The presence of incidental findings will be documented at the time of the initial CT coronary angiography and any further investigations be documented by review of the participant’s medical records.

#### Data analysis

The trial results will be reported in accordance with the CONSORT guidelines and, where possible, the clinical profile of unrecruited and ineligible patients will be recorded.

#### Statistical analysis

The trial statistician will supervise statistical analyses performed by Edinburgh Clinical Trials Unit. A full statistical analysis plan will be written separately.

#### Sample size

Previous studies have diagnosed angina pectoris in 27.0% of clinic attendees [6]. Whilst CT coronary angiography may reduce ‘false-positive’ diagnoses of angina; this intervention is most likely to increase the diagnosis of angina, given that current standard diagnostic approaches tend to be conservative and under-diagnose coronary heart disease [6]. We believe that, to be clinically useful, this intervention should increase the clinical diagnosis of angina due to coronary heart disease in at least 1 in 25 clinic attendees. For 80% power at a two-sided *P* value of 0.05, we will need to recruit 2,069 patients per group to detect an absolute increase of 4.0% in the diagnosis of angina.

#### Sample size: long-term outcome

Establishing the diagnosis of angina is an important aspect for the trial but ultimately it is the long-term patient outcome that is important; can patients’ chest pain symptoms be resolved and their long-term outcome improved? After 5 years of follow-up, we would anticipate a coronary event rate (coronary heart disease death or acute coronary syndrome) of 13.1% for the total population [6]. The study would have 80% power at a two-sided *P* value of 0.05 to detect a decrease of 2.8% in the 5-year event rate. This would also provide ~600 events and permit the exploration of up to 60 variables [28] in evaluating the predictive value of established risk factors and the novel risk factors of coronary artery calcium score and CT coronary angiography.

#### Statistical analysis plan

Where appropriate, two main comparisons will be made: (a) between the scanned and unscanned groups; and (b) in the scanned group only, between the initial clinic assessment and the final assessment following knowledge of the CT scan. The primary analysis will be a comparison between the scanned and unscanned groups, of the proportion of patients diagnosed with angina pectoris secondary to coronary heart disease at 6 weeks. This comparison will be performed using logistic regression, adjusted for the variables in the minimization algorithm.

#### Health economic analysis

Health service costs will be assigned to the type and intensity of resource use, measured by the number of diagnostic and therapeutic procedures or interventions, medications, hospital clinic attendances and hospitalization episodes from randomization to 6 months of follow-up. Costs will be attributed to the need for (i) additional invasive or noninvasive imaging, (ii) drug therapy, (iii) coronary revascularization, and (iv) hospitalization for chest pain.

Unit costs will reflect a mixture of approaches, including activity-based analyses of resource consumption for specific procedures or interventions alongside average per diem in-patient costs calculated on a specialty-specific basis using the Scottish Health Service Costs system. Centre-specific costs for imaging and revascularization procedures/interventions will be determined on the basis of measured procedure duration and the unit costs of these resources for cardiac catheterization laboratories and theatres. Local unit costs for labour, consumables, overheads and depreciation will be obtained from the finance department in each centre. The costs of novel cardiac imaging modalities will be determined using standard ‘bottom-up’ cost-accounting methods. Costs of hospital admission and out-patient visits will be measured using a ‘top-down’ costing method. These costs will be estimated for each patient in the trial using centre and specialty-specific average costs, which will also be applied to subsequent in-patient episodes and out-patient attendances beyond 6 months.

The SF-12® and SAQ-UK will be administered by self-completed postal questionnaire at baseline and at 6 weeks and 6 months of follow-up. Standard scoring algorithms will be used to calculate the SF-12® health domain profile scales and physical and mental health summary measures (PCS-12 and MCS-12, respectively). The Short Form 6D (SF-6D), a single index preference-based measure, will be calculated from the SF-12 responses using the Brazier algorithm. Missing hospital cost and quality of life data will be analyzed using multiple imputation techniques.

Cost-effectiveness will be estimated using a prospective within-trial analysis of treatment effects analyzed on an intention-to-treat basis and a decision model of long-term costs and health outcomes. Analysis will be performed from the perspective of the health care system for resource use and the individual patient for health outcomes. The primary endpoint for the economic analysis will be incremental cost-effectiveness ratios comparing the alternative diagnostic strategies’ impact on health service use and health related quality of life. The cost-effectiveness analysis will be reported in terms of the incremental cost per quality-adjusted life year (QALY) gained.

The SF-6D utility scores will be combined with survival times to enable estimation of QALYs for all randomized patients. These will be estimated within-trial and over the patients’ lifetime, by taking the sum of life years obtained in each arm of the trial within the 6-month follow-up period, modelling subsequent life expectancy and then adjusting expected life-times for observed and modelled SF-6D trajectories.

Information pertaining to resource use, cost, outcome and cost-effectiveness will be reported as the mean per patient in each arm of the trial and the mean difference, with appropriate measures of variance. Cost-effectiveness in sub-groups will be estimated by applying any reduction in overall relative risk or cost to different baseline absolute risk groups. Cost-effectiveness acceptability curves and net benefit statistics will also be reported.

Within-trial analyses will be integrated into a decision model of long-term costs and health effects. The general methods used will follow those defined as good practice by the National Institute for Health and Clinical Excellence (NICE). We propose to use a Monte-Carlo microsimulation model, similar to that recently used to assess the cost-effectiveness of 64-MDCT coronary angiography based triage for patients with low-risk chest pain [29]. Base case analysis, using the intention-to-treat results, and sensitivity analysis will be conducted by varying key model parameters and critical assumptions over plausible ranges and distributions. The decision model will also permit the analysis of cost-effectiveness, conditional on pre-specified coronary artery disease risk strata [30] or other important patient characteristics.

#### Study monitoring

The trial steering committee and trial management group include representatives from the grant applicants and trial management, as well as individuals not directly involved in the trial. The study is assessing a diagnostic intervention that is unlikely to lead to a major or safety-event threshold before the end of trial recruitment. Therefore, a data and safety monitoring committee will not be convened.

## Discussion

The multicentre randomized controlled SCOT-HEART trial will assess the added value of CT imaging in over 4,000 patients attending rapid access chest pain clinics. This will define the most appropriate use of this emerging technology in the setting of diagnosing and treating patients with coronary heart disease and angina pectoris. This study will also lay the foundation for future studies to look at the potential prognostic value of this technology.

Current opinion suggests that the main utility of CT coronary angiography lies with the diagnosis and management of patients at intermediate risk of coronary heart disease [12,20] although recent evidence suggests that it may have a role in the care of patients with suspected acute coronary syndrome [31]. However, it could have a role even in apparently high-risk patients with stable symptoms, since CT may help identify patients with ‘false-positive’ stress tests that could potentially avoid invasive coronary angiography. Alternatively, it may allow early identification of patients at high risk of invasive coronary angiography (such as critical left main stem stenosis), those with anomalous coronary anatomy, or those for whom coronary revascularization is likely to be necessary. In the latter scenario, this will facilitate planning of invasive angiography and allow an interventional cardiologist to undertake follow-on percutaneous coronary intervention as required. This would potentially avoid the need for recurrent invasive angiography in situations where a noninterventional cardiologist performs the diagnostic angiogram.

For apparently low-risk patients, the use of CT coronary angiography is controversial because of the high radiation doses and low pre-test likelihood of disease. This is a particular issue for younger patients and women [25]. However, modern scanners have dramatically reduced radiation exposure, whether because of the increased speed of multidetector scanners that capture the information in a single rotation or through the use of pulse sequences with prospective electrocardiographic gating. This may lead to better focused utilization of secondary preventative therapies in individuals who would otherwise not receive treatment. There is also the added value of imaging noncardiac structures that might be the origin of the presenting chest pain. Finally, this procedure is likely to provide more firm reassurance and potentially more rapid resolution of symptoms in patients anxious to establish whether they have significant coronary heart disease.

Economic evaluation will assist policy makers in deciding whether there is a cost-effective benefit associated with MDCT scans. This is an expensive technology and its healthcare value needs to be established. Potential benefits of MDCT lie in (i) reducing further noninvasive and invasive investigations, (ii) reducing symptoms and improving quality of life through more focused therapy, and (iii) improving long-term clinical outcomes. Thus, measurement of cost-effectiveness requires estimation of resource utilization, quality of life for all patients, and in the subsequent follow-up study, event-free survival.

Without a comprehensive assessment of all patients attending a rapid access chest pain clinic, the utility of CT will remain undefined and open to question. We believe that this requires a randomized controlled trial to evaluate this emerging and promising imaging technology in a comprehensive and pragmatic manner.

### Trial status

The study has been approved by the South East Scotland Research Ethics Committee. Recruitment is underway at eight sites. At present, 1000 participants have been recruited to the SCOT-HEART study.

## Abbreviations

AU: Agatston units; CT: Computed tomography; HDL: High-density lipoprotein; MDCT: Multidetector computed tomography; NICE: National institute of clinical excellence.

## Competing interests

The authors declare that they have no competing interests.

## Authors’ contribution

DEN conceived the study, participated in its design and coordination and helped to draft the manuscript. MCW helped to draft the manuscript. All authors read and approved the final manuscript.
